# Supplementary material for: Serum β-hCG as a Biomarker in Pancreatic Neuroendocrine Tumors: Rethinking Single-Analyte Approach
Source: Cancers (Basel). 2024 May 29;16(11):2060. doi: 10.3390/cancers16112060 (PMC11171076; doi:10.3390/cancers16112060)
Supplement: Supplementary file 1 [file cancers-16-02060-s001.zip › cancers-3003873-supplementary.pdf]

Table of Contents

1. Comparison of beta-HCG relative to Liver Tumor Burden 2
2. Comparison of beta-HCG relative to Grading 6
3. Comparison of beta-HCG relative to Treatment Response according to RECIST 1.1. 10
4. Analysis of changes in b-HCG over time 14
   a) Across whole study group 14
   b) Relative to Liver Tumor Burden=10 19
   c) Relative to Liver Tumor Burden=25 24
   d) Relative to grading=G1 28
   e) Relative to grading=G2 32
   f) Relative to Treatment Response=SD 36
   g) Relative to Treatment Response=PD 39
5. Comparison between Sample 16 and Sample 1 across study groups 43
   a) Liver Tumor Burden 44
   b) Grading 46
   c) Treatment Response 48
   d) Patient's age 51
6. Logistic regression analysis – predictive power of Initial b-HCG concentrations on the occurrence of Progressive Disease 51

1. Comparison of beta-HCG relative to Liver Tumor Burden

Supplementary Table S1.1.: Normality assessment: beta-HCG vs Liver Tumor Burden=up to 10%.

Liver Tumor Burden = up to 10%; Normality assessment (serum b-HCG)			
Variable	N	W	p
1. Sample	22	0.6918	<0.0001
2. Sample	22	0.7035	<0.0001
3. Sample	22	0.7109	<0.0001
4. Sample	22	0.7230	<0.0001
5. Sample	22	0.7316	<0.0001
6. Sample	22	0.7393	<0.0001
7. Sample	22	0.7486	<0.0001
8. Sample	22	0.7575	<0.0001
9. Sample	22	0.7725	<0.0001
10. Sample	22	0.7703	<0.0001
11. Sample	22	0.7761	<0.0001
12. Sample	22	0.7878	<0.0001
13. Sample	22	0.7821	<0.0001
14. Sample	22	0.7799	<0.0001
15. Sample	22	0.7850	<0.0001
16. Sample	22	0.7919	<0.0001

Supplementary Table S1.2.: Normality assessment: beta-HCG vs Liver Tumor Burden=10-25%.

Liver Tumor Burden = 10-25%; Normality assessment (serum b-HCG)			
Variable	N	W	p
1. Sample	18	0.9569	0.5439
2. Sample	18	0.9610	0.6220
3. Sample	18	0.9677	0.7526
4. Sample	18	0.9679	0.7583
5. Sample	18	0.9697	0.7913
6. Sample	18	0.9661	0.7210
7. Sample	18	0.9625	0.6499
8. Sample	18	0.9705	0.8068
9. Sample	18	0.9756	0.8933
10. Sample	18	0.9811	0.9604
11. Sample	18	0.9814	0.9633
12. Sample	18	0.9848	0.9860
13. Sample	18	0.9851	0.9875
14. Sample	18	0.9843	0.9832
15. Sample	18	0.9841	0.9825
16. Sample	18	0.9817	0.9658

Supplementary Table S1.3. Mann-Whitney U Test (with continuity correction): beta-HCG vs Liver Tumor Burden. Significant results are highlighted.

Lack of normal distribution, performed Mann-Whitney U Test with continuity correction.

Mann-Whitney U Test (with continuity correction) serum b-HCG vs variable: Liver Tumor Burden
Highlighted values are significant, with 2-sided $p<0.05$

Variable	Sum.rank 0.1	Sum.rank 0.25	U	Z	p	Z corrected.	p
1. Sample	275	545	22	-4.7712	<0.0001	-4.7717	<0.0001
2. Sample	272	548	19	-4.8528	<0.0001	-4.8539	<0.0001
3. Sample	271	549	18	-4.8799	<0.0001	-4.8804	<0.0001
4. Sample	271	549	18	-4.8799	<0.0001	-4.8804	<0.0001
5. Sample	271	549	18	-4.8799	<0.0001	-4.8804	<0.0001
6. Sample	270	550	17	-4.9071	<0.0001	-4.9071	<0.0001
7. Sample	271	549	18	-4.8799	<0.0001	-4.8802	<0.0001
8. Sample	268	552	15	-4.9615	<0.0001	-4.9617	<0.0001
9. Sample	269	551	16	-4.9343	<0.0001	-4.9343	<0.0001
10. Sample	269	551	16	-4.9343	<0.0001	-4.9343	<0.0001
11. Sample	269	551	16	-4.9343	<0.0001	-4.9343	<0.0001
12. Sample	269	551	16	-4.9343	<0.0001	-4.9343	<0.0001
13. Sample	271	549	18	-4.8799	<0.0001	-4.8800	<0.0001
14. Sample	271	549	18	-4.8799	<0.0001	-4.8804	<0.0001
15. Sample	272	548	19	-4.8528	<0.0001	-4.8530	<0.0001
16. Sample	272	548	19	-4.8528	<0.0001	-4.8530	<0.0001

Supplementary Table S1.4.: Descriptive statistics, beta-HCG vs Liver Tumor Burden=up to 10%.

Liver Tumor Burden = up to 10%; Descriptive statistics (serum b-HCG)								
Variable	N	Mean	Median	Minimum	Maximum	Lower Quartile	Upper Quartile	Standard Deviation
1. Sample	22	102.51	72.15	12.40	425.93	45.60	98.70	103.80
2. Sample	22	109.52	79.40	12.90	431.23	54.20	103.20	103.63
3. Sample	22	116.14	87.40	13.50	442.98	62.10	110.30	103.87
4. Sample	22	124.90	95.60	13.90	458.87	73.40	117.20	105.98
5. Sample	22	133.74	102.40	14.70	473.78	82.10	128.70	108.16
6. Sample	22	141.89	114.40	15.40	481.23	97.80	139.80	107.75
7. Sample	22	150.09	123.45	15.90	498.43	102.20	147.70	109.91
8. Sample	22	159.72	135.45	17.30	512.32	110.90	157.80	111.51
9. Sample	22	168.57	143.60	18.50	534.67	116.70	165.70	114.79
10. Sample	22	179.04	154.90	18.90	557.43	128.70	172.80	118.12
11. Sample	22	187.82	162.55	19.40	576.23	137.80	186.30	121.37
12. Sample	22	197.12	171.05	19.90	593.21	145.90	196.50	123.64
13. Sample	22	207.51	182.95	20.60	632.65	156.50	206.70	129.42
14. Sample	22	217.89	192.55	20.90	668.56	165.40	213.70	134.86
15. Sample	22	228.75	200.95	21.10	694.32	178.90	224.80	139.04
16. Sample	22	242.07	213.10	21.30	732.11	186.50	245.60	145.29

Supplementary Table S1.5.: Descriptive statistics, beta-HCG vs Liver Tumor Burden=10-25%.

Liver Tumor Burden = 10-25%; Descriptive statistics (serum b-HCG)								
Variable	N	Mean	Median	Minimum	Maximum	Lower Quartile	Upper Quartile	Standard Deviation
1. Sample	18	363.37	382.20	213.50	543.80	289.40	423.80	85.38
2. Sample	18	378.86	397.15	223.70	562.10	302.30	441.90	87.56
3. Sample	18	394.56	413.05	234.80	579.20	323.80	453.80	88.09
4. Sample	18	408.48	429.95	246.60	595.20	337.70	466.20	88.65
5. Sample	18	425.21	442.80	267.50	619.30	354.30	476.50	90.64
6. Sample	18	442.57	457.90	289.70	638.70	368.70	495.40	92.13
7. Sample	18	457.94	472.40	312.80	651.20	385.40	513.70	93.23
8. Sample	18	477.46	490.95	322.10	668.30	398.70	534.50	97.74
9. Sample	18	493.25	510.25	325.60	681.30	418.70	554.80	99.44
10. Sample	18	510.70	523.00	327.00	695.20	442.60	578.40	102.44
11. Sample	18	525.55	535.05	329.10	713.60	462.30	589.90	104.62
12. Sample	18	542.37	550.30	332.30	729.30	476.50	615.40	105.67
13. Sample	18	558.19	562.30	335.60	748.50	501.20	629.50	107.78
14. Sample	18	574.24	578.30	337.90	767.10	523.40	647.50	110.65
15. Sample	18	590.93	594.70	341.20	789.50	534.20	665.40	113.13
16. Sample	18	606.08	612.15	342.10	804.50	543.20	679.80	115.01

2. Comparison of beta-HCG relative to Grading

Supplementary Table S2.1.: Normality assessment: beta-HCG vs Grading=G1.

Grading=G1; Normality assessment (serum b-HCG)			
Variable	N	W	p
1. Sample	18	0.6261	<0.0001

Grading=G1; Normality assessment (serum b-HCG)			
Variable	N	W	p
2. Sample	18	0.6384	<0.0001
3. Sample	18	0.6428	<0.0001
4. Sample	18	0.6573	<0.0001
5. Sample	18	0.6703	<0.0001
6. Sample	18	0.6816	<0.0001
7. Sample	18	0.6887	<0.0001
8. Sample	18	0.6903	<0.0001
9. Sample	18	0.7083	0.0001
10. Sample	18	0.7066	0.0001
11. Sample	18	0.7167	<0.0001
12. Sample	18	0.7311	<0.0001
13. Sample	18	0.7307	<0.0001
14. Sample	18	0.7284	<0.0001
15. Sample	18	0.7331	<0.0001
16. Sample	18	0.7379	<0.0001

Supplementary Table S2.2.: Normality assessment: beta-HCG vs Grading=G2.

Grading=G2; Normality assessment (serum b-HCG)			
Variable	N	W	p
1. Sample	22	0.9387	0.1858
2. Sample	22	0.9394	0.1921
3. Sample	22	0.9387	0.1860
4. Sample	22	0.9344	0.1517
5. Sample	22	0.9392	0.1901
6. Sample	22	0.9396	0.1943
7. Sample	22	0.9407	0.2045

Grading=G2; Normality assessment (serum b-HCG)			
Variable	N	W	p
8. Sample	22	0.9468	0.2724
9. Sample	22	0.9451	0.2523
10. Sample	22	0.9466	0.2705
11. Sample	22	0.9454	0.2556
12. Sample	22	0.9434	0.2322
13. Sample	22	0.9409	0.2067
14. Sample	22	0.9383	0.1824
15. Sample	22	0.9364	0.1666
16. Sample	22	0.9374	0.1749

Supplementary Table S2.3.: Mann-Whitney U Test (with continuity correction): beta-HCG vs Grading. Significant results are highlighted.

Lack of normal distribution, performed Mann-Whitney U Test with continuity correction

Mann-Whitney U Test (with continuity correction) serum b-HCG vs variable: grading Highlighted values are significant, with 2-sided p<0.05							
Variable	Sum.rank G1	Sum.rank G2	U	Z	p	Z corrected	p
1. Sample	211	609	40	-4.2818	<0.0001	-4.2822	<0.0001
2. Sample	211	609	40	-4.2818	<0.0001	-4.2829	<0.0001
3. Sample	210	610	39	-4.3090	<0.0001	-4.3094	<0.0001
4. Sample	213.5	606.5	42.5	-4.2139	<0.0001	-4.2143	<0.0001
5. Sample	214	606	43	-4.2003	<0.0001	-4.2007	<0.0001
6. Sample	213	607	42	-4.2275	<0.0001	-4.2275	<0.0001
7. Sample	216	604	45	-4.1459	<0.0001	-4.1461	<0.0001
8. Sample	215	605	44	-4.1731	<0.0001	-4.1733	<0.0001
9. Sample	217	603	46	-4.1187	<0.0001	-4.1187	<0.0001
10. Sample	216	604	45	-4.1459	<0.0001	-4.1459	<0.0001
11. Sample	216	604	45	-4.1459	<0.0001	-4.1459	<0.0001

Mann-Whitney U Test (with continuity correction) serum b-HCG vs variable: grading Highlighted values are significant, with 2-sided p<0.05							
Variable	Sum.rank G1	Sum.rank G2	U	Z	p	Z corrected	p
12. Sample	215	605	44	-4.1731	<0.0001	-4.1731	<0.0001
13. Sample	216	604	45	-4.1459	<0.0001	-4.1459	<0.0001
14. Sample	217	603	46	-4.1187	<0.0001	-4.1191	<0.0001
15. Sample	218	602	47	-4.0915	<0.0001	-4.0917	<0.0001
16. Sample	216	604	45	-4.1459	<0.0001	-4.1459	<0.0001

Supplementary Table S2.4.: Descriptive statistics, beta-HCG vs Grading=G1.

Grading=G1; Descriptive statistics (serum b-HCG)								
Variable	N	Mean	Median	Minimum	Maximum	Lower Quartile	Upper Quartile	Standard Deviation
1. Sample	18	90.34	67.70	12.40	425.93	45.60	84.50	94.59
2. Sample	18	97.71	76.50	12.90	431.23	54.20	89.50	94.66
3. Sample	18	105.05	85.40	13.50	442.98	62.10	94.50	96.32
4. Sample	18	114.45	95.60	13.90	458.87	73.40	112.80	98.70
5. Sample	18	123.82	102.40	14.70	473.78	82.10	124.50	101.74
6. Sample	18	133.16	114.40	15.40	481.23	97.80	131.20	103.35
7. Sample	18	142.24	123.45	15.90	498.43	102.20	139.70	107.43
8. Sample	18	151.77	135.45	17.30	512.32	110.90	145.80	109.71
9. Sample	18	160.91	143.60	18.50	534.67	116.70	157.60	114.31
10. Sample	18	171.62	154.90	18.90	557.43	128.70	169.80	119.49
11. Sample	18	180.36	162.55	19.40	576.23	137.80	176.50	123.93
12. Sample	18	190.26	171.05	19.90	593.21	145.90	189.70	128.48
13. Sample	18	200.96	182.95	20.60	632.65	154.30	198.70	135.95
14. Sample	18	211.35	192.55	20.90	668.56	161.20	212.70	142.25
15. Sample	18	221.62	200.95	21.10	694.32	178.90	221.30	146.77
16. Sample	18	233.57	213.10	21.30	732.11	182.10	234.20	153.25

Supplementary Table S2.5.: Descriptive statistics, beta-HCG vs Grading=G2.

Grading=G2; Descriptive statistics (serum b-HCG)								
Variable	N	Mean	Median	Minimum	Maximum	Lower Quartile	Upper Quartile	Standard Deviation
1. Sample	22	325.89	354.35	34.50	543.80	287.90	423.50	123.92
2. Sample	22	339.55	368.75	39.80	562.10	293.20	439.70	127.76
3. Sample	22	353.01	375.70	45.60	579.20	298.70	452.70	130.84
4. Sample	22	365.48	390.60	49.80	595.20	304.50	462.90	133.83
5. Sample	22	380.34	409.55	57.80	619.30	308.70	476.50	137.82
6. Sample	22	395.04	427.60	69.60	638.70	324.20	493.40	141.86
7. Sample	22	408.39	442.00	76.50	651.20	342.80	512.80	145.12
8. Sample	22	426.19	457.80	86.20	668.30	367.40	531.90	150.06
9. Sample	22	440.48	473.15	94.20	681.30	378.10	546.70	153.14
10. Sample	22	456.47	480.40	112.20	695.20	389.60	564.30	156.51
11. Sample	22	470.25	488.95	126.70	713.60	402.90	584.80	159.10
12. Sample	22	485.21	507.25	142.10	729.30	415.60	597.80	161.69
13. Sample	22	499.78	522.15	156.50	748.50	428.40	623.80	164.54
14. Sample	22	514.81	545.20	165.40	767.10	440.80	641.80	167.84
15. Sample	22	530.91	570.35	174.50	789.50	453.20	664.30	170.30
16. Sample	22	546.85	593.15	186.50	804.50	469.60	675.60	170.31

3. Comparison of beta-HCG relative to Treatment Response according to RECIST 1.1.

Supplementary Table S3.1.: Normality assessment: beta-HCG vs Treatment Response=SD.

Treatment Response=SD; Normality assessment (serum b-HCG)			
Variable	N	W	p
1. Sample	19	0.6084	<0.0001

Treatment Response=SD; Normality assessment (serum b-HCG)			
Variable	N	W	p
2. Sample	19	0.6179	<0.0001
3. Sample	19	0.6222	<0.0001
4. Sample	19	0.6358	<0.0001
5. Sample	19	0.6452	<0.0001
6. Sample	19	0.6501	<0.0001
7. Sample	19	0.6580	<0.0001
8. Sample	19	0.6642	<0.0001
9. Sample	19	0.6774	<0.0001
10. Sample	19	0.6730	<0.0001
11. Sample	19	0.6774	<0.0001
12. Sample	19	0.6918	<0.0001
13. Sample	19	0.6780	<0.0001
14. Sample	19	0.6768	<0.0001
15. Sample	19	0.6850	<0.0001
16. Sample	19	0.6856	<0.0001

Supplementary Table S3.2.: Normality assessment: beta-HCG vs Treatment Response=PD.

Treatment Response=PD; Normality assessment (serum b-HCG)			
Variable	N	W	p
1. Sample	21	0.9447	0.2690
2. Sample	21	0.9469	0.2969
3. Sample	21	0.9501	0.3427
4. Sample	21	0.9491	0.3275
5. Sample	21	0.9556	0.4350
6. Sample	21	0.9574	0.4651
7. Sample	21	0.9571	0.4591
8. Sample	21	0.9656	0.6352

Treatment Response=PD; Normality assessment (serum b-HCG)			
Variable	N	W	p
9. Sample	21	0.9642	0.6052
10. Sample	21	0.9661	0.6471
11. Sample	21	0.9630	0.5793
12. Sample	21	0.9601	0.5178
13. Sample	21	0.9569	0.4562
14. Sample	21	0.9553	0.4267
15. Sample	21	0.9530	0.3868
16. Sample	21	0.9520	0.3711

Supplementary Table S3.3.: Mann-Whitney U Test (with continuity correction): beta-HCG vs Treatment Response. Significant results are highlighted.

Lack of normal distribution, performed Mann-Whitney U Test with continuity correction

Mann-Whitney U Test (with continuity correction) serum b-HCG vs variable: Treatment Response<br>Highlighted values are significant, with 2-sided p<0.05							
Variable	Sum.rank SD	Sum.rank PD	U	Z	p	Z corrected.	p
1. Sample	223	597	33	-4.4960	<0.0001	-4.4964	<0.0001
2. Sample	220	600	30	-4.5772	<0.0001	-4.5783	<0.0001
3. Sample	219	601	29	-4.6043	<0.0001	-4.6047	<0.0001
4. Sample	220	600	30	-4.5772	<0.0001	-4.5776	<0.0001
5. Sample	219	601	29	-4.6043	<0.0001	-4.6047	<0.0001
6. Sample	218	602	28	-4.6314	<0.0001	-4.6314	<0.0001
7. Sample	219	601	29	-4.6043	<0.0001	-4.6045	<0.0001
8. Sample	217	603	27	-4.6584	<0.0001	-4.6587	<0.0001
9. Sample	218	602	28	-4.6314	<0.0001	-4.6314	<0.0001
10. Sample	219	601	29	-4.6043	<0.0001	-4.6043	<0.0001
11. Sample	219	601	29	-4.6043	<0.0001	-4.6043	<0.0001
12. Sample	220	600	30	-4.5772	<0.0001	-4.5772	<0.0001
13. Sample	222	598	32	-4.5230	<0.0001	-4.5230	<0.0001

Mann-Whitney U Test (with continuity correction) serum b-HCG vs variable: Treatment Response Highlighted values are significant, with 2-sided p<0.05							
Variable	Sum.rank SD	Sum.rank PD	U	Z	p	Z corrected.	p
14. Sample	223	597	33	-4.4959	<0.0001	-4.4964	<0.0001
15. Sample	223	597	33	-4.4959	<0.0001	-4.4962	<0.0001
16. Sample	223	597	33	-4.4959	<0.0001	-4.4959	<0.0001

Supplementary Table S3.4.: Descriptive statistics, beta-HCG vs Treatment response=SD.

Treatment Response=SD; Descriptive statistics (serum b-HCG)								
Variable	N	Mean	Median	Minimum	Maximum	Lower Quartile	Upper Quartile	Standard Deviation
1. Sample	19	88.18	67.80	12.40	425.93	45.60	84.50	90.31
2. Sample	19	95.17	76.50	12.90	431.23	54.20	89.50	90.15
3. Sample	19	101.79	86.30	13.50	442.98	62.10	94.50	91.50
4. Sample	19	110.29	93.40	13.90	458.87	73.40	112.80	93.70
5. Sample	19	118.46	102.30	14.70	473.78	82.10	124.50	95.65
6. Sample	19	126.60	113.40	15.40	481.23	97.80	131.20	95.73
7. Sample	19	134.95	123.40	15.90	498.43	102.20	139.70	98.78
8. Sample	19	143.95	134.20	17.30	512.32	110.90	145.80	100.90
9. Sample	19	152.64	143.50	18.50	534.67	115.60	157.60	104.94
10. Sample	19	162.88	153.40	18.90	557.43	128.70	169.80	108.72
11. Sample	19	171.38	159.40	19.40	576.23	137.80	176.50	111.89
12. Sample	19	180.90	167.80	19.90	593.21	145.90	189.70	115.01
13. Sample	19	191.45	178.50	20.60	632.65	156.50	198.70	121.99
14. Sample	19	202.38	189.70	20.90	668.56	165.40	212.70	129.11
15. Sample	19	213.59	199.60	21.10	694.32	178.90	221.30	134.29
16. Sample	19	226.63	212.80	21.30	732.11	186.50	234.20	141.26

Supplementary Table S3.5.: Descriptive statistics, beta-HCG vs Treatment response=PD.

Treatment Response=PD; Descriptive statistics (serum b-HCG)								
Variable	N	Mean	Median	Minimum	Maximum	Lower Quartile	Upper Quartile	Standard Deviation
1. Sample	21	339.07	356.40	37.80	543.80	287.90	423.50	111.62
2. Sample	21	353.36	376.30	43.20	562.10	299.30	439.70	114.64
3. Sample	21	367.77	389.50	54.80	579.20	310.60	452.70	116.08
4. Sample	21	381.20	402.60	64.30	595.20	322.40	462.90	117.37
5. Sample	21	397.40	420.40	76.50	619.30	335.80	476.50	119.47
6. Sample	21	413.45	438.40	87.60	638.70	347.60	493.40	121.95
7. Sample	21	427.66	451.20	94.50	651.20	358.90	512.80	124.39
8. Sample	21	446.33	462.40	109.80	668.30	367.80	531.90	128.00
9. Sample	21	461.28	476.50	116.70	681.30	381.70	546.70	130.38
10. Sample	21	477.94	487.60	127.80	695.20	398.70	564.30	133.69
11. Sample	21	492.19	496.70	132.70	713.60	412.90	584.80	136.50
12. Sample	21	507.73	515.80	139.80	729.30	432.70	597.80	139.15
13. Sample	21	522.62	527.80	145.60	748.50	452.30	623.80	142.27
14. Sample	21	537.38	546.70	153.20	767.10	462.80	641.80	146.24
15. Sample	21	552.91	576.30	159.80	789.50	472.70	664.30	149.85
16. Sample	21	568.04	598.70	165.40	804.50	478.50	675.60	151.34

4. Analysis of changes in b-HCG over time

a) Across whole study group

Supplementary Table S4.1.: Normality assessment: beta-HCG changes over time across the whole group.

Normality assessment (serum b-HCG)			
Variable	N	W	p
1. Sample	40	0.8841	0.0007
2. Sample	40	0.8868	0.0008

Normality assessment (serum b-HCG)			
Variable	N	W	p
3. Sample	40	0.8877	0.0009
4. Sample	40	0.8909	0.0010
5. Sample	40	0.8955	0.0014
6. Sample	40	0.8970	0.0016
7. Sample	40	0.8997	0.0019
8. Sample	40	0.9033	0.0024
9. Sample	40	0.9059	0.0029
10. Sample	40	0.9071	0.0031
11. Sample	40	0.9087	0.0035
12. Sample	40	0.9105	0.0039
13. Sample	40	0.9113	0.0042
14. Sample	40	0.9132	0.0047
15. Sample	40	0.9147	0.0053
16. Sample	40	0.9179	0.0066

Supplementary Table S4.2a.: ANOVA Friedman test and Kendall's coefficient of concordance: beta-HCG changes over time across the whole group.

Lack of normal distribution, performed ANOVA Friedman test.

ANOVA Friedman test and Kendall's coefficient of concordance(serum b-HCG)<br>Chi squared ANOVA(N = 40, df = 15) = 600.0000<br>p =<0.0001<br>Coefficient of concordance= 1.0000 r mean. rank = 1.0000				
Variable	Mean Rank	Sum Rank	Mean	Standard Deviation
1. Sample	1	40	219.89	162.03
2. Sample	2	80	230.72	165.95
3. Sample	3	120	241.43	169.91

ANOVA Friedman test and Kendall's coefficient of concordance(serum b-HCG)<br>Chi squared ANOVA(N = 40, df = 15) = 600.0000<br>p =<0.0001<br>Coefficient of concordance= 1.0000 r mean. rank = 1.0000				
Variable	Mean Rank	Sum Rank	Mean	Standard Deviation
4. Sample	4	160	252.51	172.88
5. Sample	5	200	264.90	177.32
6. Sample	6	240	277.20	181.39
7. Sample	7	280	288.62	185.34
8. Sample	8	320	302.70	191.01
9. Sample	9	360	314.67	195.36
10. Sample	9	400	328.29	200.03
11. Sample	11	440	339.80	204.10
12. Sample	12	480	352.49	208.22
13. Sample	13	520	365.31	212.84
14. Sample	14	560	378.25	217.63
15. Sample	15	600	391.73	222.01
16. Sample	16	640	405.87	225.32

Supplementary Table S4.2b.: ANOVA Friedman test and Kendall's coefficient of concordance: beta-HCG changes over time across the whole group. Significant differences are highlighted in red.

**P-value**	1. Sample	2. Sample	3. Sample	4. Sample	5. Sample	6. Sample	7. Sample	8. Sample	9. Sample	10. Sample	11. Sample	12. Sample	13. Sample	14. Sample	15. Sample	16. Sample
1. Sample		1	1	0.5799	0.0206	0.0003	0.0002	<0.0001	<0.0001	<0.0001	<0.0001	<0.0001	<0.0001	<0.0001	<0.0001	<0.0001
2. Sample	1		1	1	0.5799	0.0206	0.0003	0.0002	<0.0001	<0.0001	<0.0001	<0.0001	<0.0001	<0.0001	<0.0001	<0.0001
3. Sample	1	1		1	1	0.5799	0.0206	0.0003	0.0002	<0.0001	<0.0001	<0.0001	<0.0001	<0.0001	<0.0001	<0.0001
4. Sample	0.5799	1	1		1	1	0.5799	0.0206	0.0003	0.0002	<0.0001	<0.0001	<0.0001	<0.0001	<0.0001	<0.0001
5. Sample	0.0206	0.5799	1	1		1	1	0.5799	0.0206	0.0003	0.0002	<0.0001	<0.0001	<0.0001	<0.0001	<0.0001
6. Sample	0.0003	0.0206	0.5799	1	1		1	1	0.5799	0.0206	0.0003	0.0002	<0.0001	<0.0001	<0.0001	<0.0001
7. Sample	<0.0001	0.0003	0.0206	0.5799	1	1		1	1	0.5799	0.0206	0.0003	0.0002	<0.0001	<0.0001	<0.0001
8. Sample	<0.0001	<0.0001	0.0003	0.0206	0.5799	1	1		1	1	0.5799	0.0206	0.0003	0.0002	<0.0001	<0.0001
9. Sample	<0.0001	<0.0001	<0.0001	0.0003	0.0206	0.5799	1	1		1	1	0.5799	0.0206	0.0003	0.0002	<0.0001
10. Sample	<0.0001	<0.0001	<0.0001	<0.0001	0.0003	0.0206	0.5799	1	1		1	1	0.5799	0.0206	0.0003	0.0002
11. Sample	<0.0001	<0.0001	<0.0001	<0.0001	<0.0001	0.0003	0.0206	0.5799	1	1		1	1	0.5799	0.0206	0.0003
12. Sample	<0.0001	<0.0001	<0.0001	<0.0001	<0.0001	<0.0001	0.0003	0.0206	0.5799	1	1		1	1	0.5799	0.0206
13. Sample	<0.0001	<0.0001	<0.0001	<0.0001	<0.0001	<0.0001	<0.0001	0.0003	0.0206	0.5799	1	1		1	1	0.5799
14. Sample	<0.0001	<0.0001	<0.0001	<0.0001	<0.0001	<0.0001	<0.0001	<0.0001	0.0003	0.0206	0.5799	1	1		1	1
15. Sample	<0.0001	<0.0001	<0.0001	<0.0001	<0.0001	<0.0001	<0.0001	<0.0001	<0.0001	0.0003	0.0206	0.5799	1	1		1
16. Sample	<0.0001	<0.0001	<0.000001	<0.0001	<0.0001	<0.0001	<0.0001	<0.0001	<0.0001	<0.0001	0.0003	0.0206	0.5799	1	1	

Supplementary Table S4.3.: Page test for trend: b-HCG changes over time across the whole group.

**Page Test for trend**	
Statistics L	59840
Statistics Z	24.4949
The one-sided p-value	<0.0001

Supplementary Table S4.4.: Descriptive statistics: b-HCG changes over time across the whole group.

Descriptive statistics (serum b-HCG)								
Variable	N	Mean	Median	Minimum	Maximum	Lower Quartile	Upper Quartile	Standard Deviation
1. Sample	40	219.89	200.55	12.40	543.80	67.70	382.20	162.03
2. Sample	40	230.72	210.20	12.90	562.10	76.50	397.15	165.95
3. Sample	40	241.43	219.65	13.50	579.20	85.40	413.05	169.91
4. Sample	40	252.51	230.20	13.90	595.20	92.85	429.95	172.88
5. Sample	40	264.90	247.55	14.70	619.30	102.05	442.80	177.32
6. Sample	40	277.20	263.20	15.40	638.70	112.85	457.90	181.39
7. Sample	40	288.62	278.95	15.90	651.20	121.00	472.40	185.34
8. Sample	40	302.70	293.75	17.30	668.30	130.90	490.95	191.01
9. Sample	40	314.67	303.95	18.50	681.30	139.55	510.25	195.36
10. Sample	40	328.29	310.10	18.90	695.20	151.05	523.00	200.03
11. Sample	40	339.80	318.30	19.40	713.60	157.90	535.05	204.10
12. Sample	40	352.49	324.95	19.90	729.30	166.05	550.30	208.22
13. Sample	40	365.31	332.15	20.60	748.50	176.50	562.30	212.84
14. Sample	40	378.25	335.20	20.90	767.10	189.70	578.30	217.63
15. Sample	40	391.73	341.50	21.10	789.50	198.60	594.70	222.01
16. Sample	40	405.87	358.85	21.30	804.50	212.60	612.15	225.32

b) Relative to Liver Tumor Burden=10

Supplementary Table S4.5.: Normality assessment: beta-HCG changes over time in the Liver Tumor Burden=10% subgroup.

Normality assessment (serum b-HCG)Considered condition: v6="0.1"			
Variable	N	W	p
1. Sample	22	0.6918	<0.0001

Normality assessment (serum b-HCG)Considered condition: v6="0.1"			
Variable	N	W	p
2. Sample	22	0.7035	<0.0001
3. Sample	22	0.7109	<0.0001
4. Sample	22	0.7230	<0.0001
5. Sample	22	0.7316	<0.0001
6. Sample	22	0.7393	<0.0001
7. Sample	22	0.7486	<0.0001
8. Sample	22	0.7575	0.0001
9. Sample	22	0.7725	0.0002
10. Sample	22	0.7704	0.0002
11. Sample	22	0.7761	0.0002
12. Sample	22	0.7878	0.0003
13. Sample	22	0.7821	0.0003
14. Sample	22	0.7799	0.0002
15. Sample	22	0.7850	0.0003
16. Sample	22	0.7918	0.0004

Supplementary Table S4.5a.: ANOVA Friedman test and Kendall's coefficient of concordance: beta-HCG changes over time in the Liver Tumor Burden=10% subgroup.

Lack of normal distribution, performed ANOVA Friedman test.

ANOVA Friedman test and Kendall's coefficient of concordance(serum b-HCG)
Chi squared. ANOVA(N = 22, df = 15) = 330.0000
p =<0.0001
Coefficient of concordance= 1.0000 r mean rank = 1.0000
Considered condition: LTB=10%

Variable	Mean Rank	Suma Rank	Mean	Standard Deviation
1. Sample	1	22	102.51	103.80
2. Sample	2	44	109.52	103.63
3. Sample	3	66	116.14	103.87
4. Sample	4	88	124.90	105.98
5. Sample	5	110	133.74	108.16
6. Sample	6	132	141.89	107.75
7. Sample	7	154	150.09	109.91
8. Sample	8	176	159.72	111.51
9. Sample	9	198	168.57	114.79
10. Sample	10	220	179.04	118.12
11. Sample	11	242	187.82	121.37
12. Sample	12	264	197.12	123.64
13. Sample	13	286	207.51	129.42
14. Sample	14	308	217.89	134.86
15. Sample	15	330	228.75	139.04
16. Sample	16	352	242.07	145.29

Supplementary Table S4.5b.: ANOVA Friedman test and Kendall's coefficient of concordance: beta-HCG changes over time in the Liver Tumor Burden=10% subgroup.

**P-value**	1. Sample	2. Sample	3. Sample	4. Sample	5. Sample	6. Sample	7. Sample	8. Sample	9. Sample	10. Sample	11. Sample	12. Sample	13. Sample	14. Sample	15. Sample	16. Sample
1. Sample		1	1	1	0.6393	0.0595	0.0035	0.0001	0.0001	<0.0001	<0.0001	<0.0001	<0.0001	<0.0001	<0.0001	<0.0001
2. Sample	1		1	1	1	0.6393	0.0595	0.0035	0.0001	0.0001	<0.0001	<0.0001	<0.0001	<0.0001	<0.0001	<0.0001
3. Sample	1	1		1	1	1	0.6393	0.0595	0.0035	0.0001	0.0001	<0.0001	<0.0001	<0.0001	<0.0001	<0.0001
4. Sample	1	1	1		1	1	1	0.6393	0.0595	0.0035	0.0001	0.0001	<0.0001	<0.0001	<0.0001	<0.0001
5. Sample	0.6393	1	1	1		1	1	1	0.6393	0.0595	0.0035	0.0001	0.0001	<0.0001	<0.0001	<0.0001
6. Sample	0.0595	0.6393	1	1	1		1	1	1	0.6393	0.0595	0.0035	0.0001	0.0001	<0.0001	<0.0001
7. Sample	0.0035	0.0595	0.6393	1	1	1		1	1	1	0.6393	0.0595	0.0035	0.0001	0.0001	<0.0001
8. Sample	0.0001	0.0035	0.0595	0.6393	1	1	1		1	1	1	0.6393	0.0595	0.0035	0.0001	0.0001
9. Sample	<0.0001	0.0001	0.0035	0.0595	0.6393	1	1	1		1	1	1	0.6393	0.0595	0.0035	0.0001
10. Sample	<0.0001	<0.0001	0.0001	0.0035	0.0595	0.6393	1	1	1		1	1	1	0.6393	0.0595	0.0035
11. Sample	<0.0001	<0.0001	<0.0001	0.0001	0.0035	0.0595	0.6393	1	1	1		1	1	1	0.6393	0.0595
12. Sample	<0.0001	<0.0001	<0.0001	<0.0001	0.0001	0.0035	0.0595	0.6393	1	1	1		1	1	1	0.6393
13. Sample	<0.0001	<0.0001	<0.0001	<0.0001	<0.0001	0.0001	0.0035	0.0595	0.6393	1	1	1		1	1	1
14. Sample	<0.0001	<0.0001	<0.0001	<0.0001	<0.0001	<0.0001	0.0001	0.0035	0.0595	0.6393	1	1	1		1	1
15. Sample	<0.0001	<0.0001	<0.0001	<0.0001	<0.0001	<0.0001	<0.0001	0.0001	0.0035	0.0595	0.6393	1	1	1		1
16. Sample	<0.0001	<0.0001	<0.0001	<0.0001	<0.0001	<0.0001	<0.0001	<0.0001	0.0001	0.0035	0.0595	0.6393	1	1	1	

Supplementary Table S4.6.: Page test for trend: b-HCG changes over time in the Liver Tumor Burden=10% subgroup.

**Page Test for trend**	
Statistics L	32912
Statistics Z	18.1659
The one-sided p-value	<0.0001

Supplementary Table S4.7.: Descriptive statistics: b-HCG changes over time in the Liver Tumor Burden=10% subgroup

Descriptive statistics (serum b-HCG)Considered condition: LTB=10%								
Variable	N	Mean	Median	Minimum	Maximum	Lower Quartile	Upper Quartile	Standard Deviation
1. Sample	22	102.51	72.15	12.40	425.93	45.60	98.70	103.80
2. Sample	22	109.52	79.40	12.90	431.23	54.20	103.20	103.63
3. Sample	22	116.14	87.40	13.50	442.98	62.10	110.30	103.87
4. Sample	22	124.90	95.60	13.90	458.87	73.40	117.20	105.98
5. Sample	22	133.74	102.40	14.70	473.78	82.10	128.70	108.16
6. Sample	22	141.89	114.40	15.40	481.23	97.80	139.80	107.75
7. Sample	22	150.09	123.45	15.90	498.43	102.20	147.70	109.91
8. Sample	22	159.72	135.45	17.30	512.32	110.90	157.80	111.51
9. Sample	22	168.57	143.60	18.50	534.67	116.70	165.70	114.79
10. Sample	22	179.04	154.90	18.90	557.43	128.70	172.80	118.12
11. Sample	22	187.82	162.55	19.40	576.23	137.80	186.30	121.37
12. Sample	22	197.12	171.05	19.90	593.21	145.90	196.50	123.64
13. Sample	22	207.51	182.95	20.60	632.65	156.50	206.70	129.42
14. Sample	22	217.89	192.55	20.90	668.56	165.40	213.70	134.86
15. Sample	22	228.75	200.95	21.10	694.32	178.90	224.80	139.04
16. Sample	22	242.07	213.10	21.30	732.11	186.50	245.60	145.29

c) Relative to Liver Tumor Burden=25

Normality assessment

Supplementary Table S4.8.: Normality assessment: beta-HCG changes over time in the Liver Tumor Burden=10-25% subgroup.

Normality assessment (serum b-HCG)Considered condition: LTB=10-25%			
Variable	N	W	p
1. Sample	18	0.9569	0.5439
2. Sample	18	0.9610	0.6220
3. Sample	18	0.9677	0.7526
4. Sample	18	0.9680	0.7583
5. Sample	18	0.9697	0.7913
6. Sample	18	0.9661	0.7210
7. Sample	18	0.9625	0.6499
8. Sample	18	0.9705	0.8068
9. Sample	18	0.9756	0.8933
10. Sample	18	0.9811	0.9604
11. Sample	18	0.9814	0.9633
12. Sample	18	0.9848	0.9860
13. Sample	18	0.9851	0.9875
14. Sample	18	0.9843	0.9832
15. Sample	18	0.9841	0.9825
16. Sample	18	0.9817	0.9658

Supplementary Table S4.8a.: ANOVA Friedman test and Kendall's coefficient of concordance: beta-HCG changes over time in the Liver Tumor Burden=10-25% subgroup.

Lack of normal distribution, performed ANOVA Friedman test.

ANOVA Friedman test and Kendall's coefficient of concordance(serum b-HCG)
Chi squared. ANOVA(N = 18, df = 15) = 270.0000
p =<0.0001
Coefficient of concordance= 1.0000 r mean. rank = 1.0000
Considered condition: LTB=10-25%

Variable	Mean Rank	Sum Rank	Mean	Standard Deviation
1. Sample	1	18	363.37	85.38
2. Sample	2	36	378.86	87.56
3. Sample	3	54	394.56	88.09
4. Sample	4	72	408.48	88.65
5. Sample	5	90	425.21	90.64
6. Sample	6	108	442.57	92.13
7. Sample	7	126	457.94	93.23
8. Sample	8	144	477.46	97.74
9. Sample	9	162	493.25	99.44
10. Sample	10	180	510.70	102.44
11. Sample	11	198	525.55	104.62
12. Sample	12	216	542.37	105.67
13. Sample	13	234	558.19	107.78
14. Sample	14	252	574.24	110.65
15. Sample	15	270	590.93	113.13
16. Sample	16	288	606.08	115.01

Supplementary Table S4.8b.: ANOVA Friedman test and Kendall's coefficient of concordance: beta-HCG changes over time in the Liver Tumor Burden=10-25% subgroup. Significant differences are highlighted in red.

**P-value**	1. Sample	2. Sample	3. Sample	4. Sample	5. Sample	6. Sample	7. Sample	8. Sample	9. Sample	10. Sample	11. Sample	12. Sample	13. Sample	14. Sample	15. Sample	16. Sample
1. Sample		1	1	1	1	0.1955	0.0188	0.0012	<0.0001	<0.0001	<0.0001	<0.0001	<0.0001	<0.0001	<0.0001	<0.0001
2. Sample	1		1	1	1	1	0.1955	0.0188	0.0012	<0.0001	<0.0001	<0.0001	<0.0001	<0.0001	<0.0001	<0.0001
3. Sample	1	1		1	1	1	1	0.1955	0.0188	0.0012	<0.0001	<0.0001	<0.0001	<0.0001	<0.0001	<0.0001
4. Sample	1	1	1		1	1	1	1	0.1955	0.0188	0.0012	<0.0001	<0.0001	<0.0001	<0.0001	<0.0001
5. Sample	1	1	1	1		1	1	1	1	0.1955	0.0188	0.0012	<0.0001	<0.0001	<0.0001	<0.0001
6. Sample	0.1955	1	1	1	1		1	1	1	1	0.1955	0.0188	0.0012	<0.0001	<0.0001	<0.0001
7. Sample	0.0188	0.1955	1	1	1	1		1	1	1	1	0.1955	0.0188	0.0012	<0.0001	<0.0001
8. Sample	0.0012	0.0188	0.1955	1	1	1	1		1	1	1	1	0.1955	0.0188	0.0012	<0.0001
9. Sample	<0.0001	0.0012	0.0188	0.1955	1	1	1	1		1	1	1	1	0.1955	0.0188	0.0012
10. Sample	<0.0001	<0.0001	0.0012	0.0188	0.1955	1	1	1	1		1	1	1	1	0.1955	0.0188
11. Sample	<0.0001	<0.0001	<0.0001	0.0012	0.0188	0.1955	1	1	1	1		1	1	1	1	0.1955
12. Sample	<0.0001	<0.0001	<0.0001	<0.0001	0.0012	0.0188	0.1955	1	1	1	1		1	1	1	1
13. Sample	<0.0001	<0.0001	<0.0001	<0.0001	<0.0001	0.0012	0.0188	0.1955	1	1	1	1		1	1	1
14. Sample	<0.0001	<0.0001	<0.0001	<0.0001	<0.0001	<0.0001	0.0012	0.0188	0.1955	1	1	1	1		1	1
15. Sample	<0.0001	<0.0001	<0.0001	<0.0001	<0.0001	<0.0001	<0.0001	0.0012	0.0188	0.1955	1	1	1	1		1
16. Sample	<0.0001	<0.0001	<0.0001	<0.0001	<0.0001	<0.0001	<0.0001	<0.0001	0.0012	0.0188	0.1955	1	1	1	1	

Supplementary Table S4.9.: Page test for trend: b-HCG changes over time in the Liver Tumor Burden=10-25% subgroup.

**Page Test for trend**	
Statistics L	26928
Statistics Z	16.4317
The one-sided p-value	<0.0001

Supplementary Table S4.10.: Descriptive statistics: b-HCG changes over time in the Liver Tumor Burden=10% subgroup.

Descriptive statistics (serum b-HCG)Considered condition: v6="0.25"								
Variable	N	Mean	Median	Minimum	Maximum	Lower Quartile	Upper Quartile	Standard Deviation
1. Sample	18	363.37	382.20	213.50	543.80	289.40	423.80	85.38
2. Sample	18	378.86	397.15	223.70	562.10	302.30	441.90	87.56
3. Sample	18	394.56	413.05	234.80	579.20	323.80	453.80	88.09
4. Sample	18	408.48	429.95	246.60	595.20	337.70	466.20	88.65
5. Sample	18	425.21	442.80	267.50	619.30	354.30	476.50	90.64
6. Sample	18	442.57	457.90	289.70	638.70	368.70	495.40	92.13
7. Sample	18	457.94	472.40	312.80	651.20	385.40	513.70	93.23
8. Sample	18	477.46	490.95	322.10	668.30	398.70	534.50	97.74
9. Sample	18	493.25	510.25	325.60	681.30	418.70	554.80	99.44
10. Sample	18	510.70	523.00	327.00	695.20	442.60	578.40	102.44
11. Sample	18	525.55	535.05	329.10	713.60	462.30	589.90	104.62
12. Sample	18	542.37	550.30	332.30	729.30	476.50	615.40	105.67
13. Sample	18	558.19	562.30	335.60	748.50	501.20	629.50	107.78
14. Sample	18	574.24	578.30	337.90	767.10	523.40	647.50	110.65
15. Sample	18	590.93	594.70	341.20	789.50	534.20	665.40	113.13
16. Sample	18	606.08	612.15	342.10	804.50	543.20	679.80	115.01

d) Relative to grading=G1

Supplementary Table S4.11.: Normality assessment: beta-HCG changes over time in the Grading=G1 subgroup.

Normality assessment (serum b-HCG)Considered condition: Grading=G1			
Variable	N	W	p
1. Sample	18	0.6261	<0.0001
2. Sample	18	0.6384	<0.0001
3. Sample	18	0.6428	<0.0001
4. Sample	18	0.6573	<0.0001
5. Sample	18	0.6703	<0.0001
6. Sample	18	0.6816	<0.0001
7. Sample	18	0.6887	<0.0001
8. Sample	18	0.6903	<0.0001
9. Sample	18	0.7083	0.0001
10. Sample	18	0.7066	<0.0001
11. Sample	18	0.7167	0.0001
12. Sample	18	0.7311	0.0002
13. Sample	18	0.7307	0.0002
14. Sample	18	0.7284	0.0002
15. Sample	18	0.7331	0.0002
16. Sample	18	0.7379	0.0002

Supplementary Table S4.12a.: ANOVA Friedman test and Kendall's coefficient of concordance: beta-HCG changes over time in the Grading=G1 subgroup.

Lack of normal distribution, performed ANOVA Friedman test.

ANOVA Friedman test and Kendall's coefficient of concordance(serum b-HCG)<br>Chi squared. ANOVA(N = 18, df = 15) = 270,0000<br>p =<0.0001<br>Coefficient of concordance= 1.0000 r mean. rank = 1.0000<br>Considered condition: Grading=G1				
Variable	Mean Rank	Sum Rank	Mean	Standard Deviation
1. Sample	18	18	90.34	94.59
2. Sample	36	36	97.71	94.66
3. Sample	54	54	105.05	96.32
4. Sample	72	72	114.45	98.70
5. Sample	90	90	123.82	101.74
6. Sample	108	108	133.16	103.35
7. Sample	126	126	142.24	107.43
8. Sample	144	144	151.77	109.71
9. Sample	162	162	160.91	114.31
10. Sample	180	180	171.62	119.49
11. Sample	198	198	180.36	123.93
12. Sample	216	216	190.26	128.48
13. Sample	234	234	200.96	135.95
14. Sample	252	252	211.35	142.25
15. Sample	270	270	221.62	146.77
16. Sample	288	288	233.57	153.25

Supplementary Table S4.12b.: ANOVA Friedman test and Kendall's coefficient of concordance: beta-HCG changes over time in the Grading=G1 subgroup. Significant results are highlighted in red.

**P-value**	1. Sample	2. Sample	3. Sample	4. Sample	5. Sample	6. Sample	7. Sample	8. Sample	9. Sample	10. Sample	11. Sample	12. Sample	13. Sample	14. Sample	15. Sample	16. Sample
1. Sample		1	1	1	1	0.1955	0.0188	0.0012	<0.0001	<0.0001	<0.0001	<0.0001	<0.0001	<0.0001	<0.0001	<0.0001
2. Sample	1		1	1	1	1	0.1955	0.0188	0.0012	<0.0001	<0.0001	<0.0001	<0.0001	<0.0001	<0.0001	<0.0001
3. Sample	1	1		1	1	1	1	0.1955	0.0188	0.0012	<0.0001	<0.0001	<0.0001	<0.0001	<0.0001	<0.0001
4. Sample	1	1	1		1	1	1	1	0.1955	0.0188	0.0012	<0.0001	<0.0001	<0.0001	<0.0001	<0.0001
5. Sample	1	1	1	1		1	1	1	1	0.1955	0.0188	0.0012	<0.0001	<0.0001	<0.0001	<0.0001
6. Sample	0.1955	1	1	1	1		1	1	1	1	0.1955	0.0188	0.0012	<0.0001	<0.0001	<0.0001
7. Sample	0.0188	0.1955	1	1	1	1		1	1	1	1	0.1955	0.0188	0.0012	<0.0001	<0.0001
8. Sample	0.0012	0.0188	0.1955	1	1	1	1		1	1	1	1	0.1955	0.0188	0.0012	<0.0001
9. Sample	<0.0001	0.0012	0.0188	0.1955	1	1	1	1		1	1	1	1	0.1955	0.0188	0.0012
10. Sample	<0.0001	<0.0001	0.0012	0.0188	0.1955	1	1	1	1		1	1	1	1	0.1955	0.0188
11. Sample	<0.0001	<0.0001	<0.0001	0.0012	0.0188	0.1955	1	1	1	1		1	1	1	1	0.1955
12. Sample	<0.0001	<0.0001	<0.0001	<0.0001	0.0012	0.0188	0.1955	1	1	1	1		1	1	1	1
13. Sample	<0.0001	<0.0001	<0.0001	<0.0001	<0.0001	0.0012	0.0188	0.1955	1	1	1	1		1	1	1
14. Sample	<0.0001	<0.0001	<0.0001	<0.0001	<0.0001	<0.0001	0.0012	0.0188	0.1955	1	1	1	1		1	1
15. Sample	<0.0001	<0.0001	<0.0001	<0.0001	<0.0001	<0.0001	<0.0001	0.0012	0.0188	0.1955	1	1	1	1		1
16. Sample	<0.0001	<0.0001	<0.0001	<0.0001	<0.0001	<0.0001	<0.0001	<0.0001	0.0012	0.0188	0.1955	1	1	1	1	

Supplementary Table S4.13.: Page test for trend: b-HCG changes over time in the Grading=G1 subgroup.

**Page Test for trend**	
Statistics L	26928
Statistics Z	16.4317
The one-sided p-value	<0.0001

Supplementary Table S4.14.: Descriptive statistics: b-HCG changes over time in the Grading=G1 subgroup.

Descriptive statistics (serum b-HCG)Considered condition: Grading="G1"								
Variable	N	Mean	Median	Minimum	Maximum	Lower Quartile	Upper Quartile	Standard Deviation
1. Sample	18	90.34	67.70	12.40	425.93	45.60	84.50	94.59
2. Sample	18	97.71	76.50	12.90	431.23	54.20	89.50	94.66
3. Sample	18	105.05	85.40	13.50	442.98	62.10	94.50	96.32
4. Sample	18	114.45	95.60	13.90	458.87	73.40	112.80	98.70
5. Sample	18	123.82	102.40	14.70	473.78	82.10	124.50	101.74
6. Sample	18	133.16	114.40	15.40	481.23	97.80	131.20	103.35
7. Sample	18	142.24	123.45	15.90	498.43	102.20	139.70	107.43
8. Sample	18	151.77	135.45	17.30	512.32	110.90	145.80	109.71
9. Sample	18	160.91	143.60	18.50	534.67	116.70	157.60	114.31
10. Sample	18	171.62	154.90	18.90	557.43	128.70	169.80	119.49
11. Sample	18	180.36	162.55	19.40	576.23	137.80	176.50	123.93
12. Sample	18	190.26	171.05	19.90	593.21	145.90	189.70	128.48
13. Sample	18	200.96	182.95	20.60	632.65	154.30	198.70	135.95
14. Sample	18	211.35	192.55	20.90	668.56	161.20	212.70	142.25
15. Sample	18	221.62	200.95	21.10	694.32	178.90	221.30	146.77

Descriptive statistics (serum b-HCG)Considered condition: Grading="G1"								
Variable	N	Mean	Median	Minimum	Maximum	Lower Quartile	Upper Quartile	Standard Deviation
16. Sample	18	233.57	213.10	21.30	732.11	182.10	234.20	153.25

e) Relative to grading=G2

Supplementary Table S4.15.: Normality assessment: beta-HCG changes over time in the Grading=G2 subgroup.

Normality assessment (serum b-HCG)Considered condition: Grading=G2			
Variable	N	W	p
1. Sample	22	0.9387	0.1858
2. Sample	22	0.9394	0.1921
3. Sample	22	0.9387	0.1860
4. Sample	22	0.9344	0.1517
5. Sample	22	0.9392	0.1901
6. Sample	22	0.9396	0.1943
7. Sample	22	0.9407	0.2045
8. Sample	22	0.9468	0.2724
9. Sample	22	0.9451	0.2523
10. Sample	22	0.9466	0.2705
11. Sample	22	0.9454	0.2556
12. Sample	22	0.9434	0.2322
13. Sample	22	0.9409	0.2067
14. Sample	22	0.9383	0.1824
15. Sample	22	0.9364	0.1666
16. Sample	22	0.9374	0.1749

Supplementary Table S4.16a.: ANOVA Friedman test and Kendall's coefficient of concordance: beta-HCG changes over time in the Grading=G2 subgroup.

Lack of normal distribution, performed ANOVA Friedman test.

ANOVA Friedman test and Kendall's coefficient of concordance(serum b-HCG)<br>Chi squared. ANOVA(N = 22, df = 15) = 330.0000<br>p =<0.0001<br>Coefficient of concordance= 1.0000 r mean. rank = 1.0000<br>Considered condition: Grading=G2				
Variable	Mean Rank	Suma Rank	Mean	Standard Deviation
1. Sample	1	22	325.89	123.92
2. Sample	2	44	339.55	127.76
3. Sample	3	66	353.01	130.84
4. Sample	4	88	365.48	133.83
5. Sample	5	110	380.34	137.82
6. Sample	6	132	395.04	141.86
7. Sample	7	154	408.39	145.12
8. Sample	8	176	426.19	150.06
9. Sample	9	198	440.48	153.14
10. Sample	10	220	456.47	156.51
11. Sample	11	242	470.25	159.10
12. Sample	12	264	485.21	161.69
13. Sample	13	286	499.78	164.54
14. Sample	14	308	514.81	167.84
15. Sample	15	330	530.91	170.30
16. Sample	16	352	546.85	170.31

Supplementary Table S4.16b.: ANOVA Friedman test and Kendall's coefficient of concordance: beta-HCG changes over time in the Grading=G2 subgroup. Significant differences are highlighted in red.

**P-value**	1. Sample	2. Sample	3. Sample	4. Sample	5. Sample	6. Sample	7. Sample	8. Sample	9. Sample	10. Sample	11. Sample	12. Sample	13. Sample	14. Sample	15. Sample	16. Sample
1. Sample		1	1	1	0.6393	0.0595	0.0035	0.0001	<0.0001	<0.0001	<0.0001	<0.0001	<0.0001	<0.0001	<0.0001	<0.0001
2. Sample	1		1	1	1	0.6393	0.0595	0.0035	0.0001	<0.0001	<0.0001	<0.0001	<0.0001	<0.0001	<0.0001	<0.0001
3. Sample	1	1		1	1	1	0.6393	0.0595	0.0035	0.0001	<0.0001	<0.0001	<0.0001	<0.0001	<0.0001	<0.0001
4. Sample	1	1	1		1	1	1	0.6393	0.0595	0.0035	0.0001	<0.0001	<0.0001	<0.0001	<0.0001	<0.0001
5. Sample	0.6393	1	1	1		1	1	1	0.6393	0.0595	0.0035	0.0001	<0.0001	<0.0001	<0.0001	<0.0001
6. Sample	0.0595	0.6393	1	1	1		1	1	1	0.6393	0.0595	0.0035	0.0001	<0.0001	<0.0001	<0.0001
7. Sample	0.0035	0.0595	0.6393	1	1	1		1	1	1	0.6393	0.0595	0.0035	0.0001	<0.0001	<0.0001
8. Sample	0.0001	0.0035	0.0595	0.6393	1	1	1		1	1	1	0.6393	0.0595	0.0035	0.0001	<0.0001
9. Sample	<0.0001	0.0001	0.0035	0.0595	0.6393	1	1	1		1	1	1	0.6393	0.0595	0.0035	0.0001
10. Sample	<0.0001	<0.0001	0.0001	0.0035	0.0595	0.6393	1	1	1		1	1	1	0.6393	0.0595	0.0035
11. Sample	<0.0001	<0.0001	<0.0001	0.0001	0.0035	0.0595	0.6393	1	1	1		1	1	1	0.6393	0.0595
12. Sample	<0.0001	<0.0001	<0.0001	<0.0001	0.0001	0.0035	0.0595	0.6393	1	1	1		1	1	1	0.6393
13. Sample	<0.0001	<0.0001	<0.0001	<0.0001	<0.0001	0.0001	0.0035	0.0595	0.6393	1	1	1		1	1	1
14. Sample	<0.0001	<0.0001	<0.0001	<0.0001	<0.0001	<0.0001	0.0001	0.0035	0.0595	0.6393	1	1	1		1	1
15. Sample	<0.0001	<0.0001	<0.0001	<0.0001	<0.0001	<0.0001	<0.0001	0.0001	0.0035	0.0595	0.6393	1	1	1		1
16. Sample	<0.0001	<0.0001	<0.0001	<0.0001	<0.0001	<0.0001	<0.0001	<0.0001	0.0001	0.0035	0.0595	0.6393	1	1	1	

Supplementary Table S4.17.: Page test for trend: b-HCG changes over time in the Grading=G2 subgroup.

**Page Test for trend**	
Statistics L	32912
Statistics Z	18.1659
The one-sided p-value	<0.0001

Supplementary Table S4.18.: Descriptive statistics: b-HCG changes over time in the Grading=G2 subgroup.

Descriptive statistics (serum b-HCG)Considered condition: Grading=G2								
Variable	N	Mean	Median	Minimum	Maximum	Lower Quartile	Upper Quartile	Standard Deviation
1. Sample	22	325.89	354.35	34.50	543.80	287.90	423.50	123.92
2. Sample	22	339.55	368.75	39.80	562.10	293.20	439.70	127.76
3. Sample	22	353.01	375.70	45.60	579.20	298.70	452.70	130.84
4. Sample	22	365.48	390.60	49.80	595.20	304.50	462.90	133.83
5. Sample	22	380.34	409.55	57.80	619.30	308.70	476.50	137.82
6. Sample	22	395.04	427.60	69.60	638.70	324.20	493.40	141.86
7. Sample	22	408.39	442.00	76.50	651.20	342.80	512.80	145.12
8. Sample	22	426.19	457.80	86.20	668.30	367.40	531.90	150.06
9. Sample	22	440.48	473.15	94.20	681.30	378.10	546.70	153.14
10. Sample	22	456.47	480.40	112.20	695.20	389.60	564.30	156.51
11. Sample	22	470.25	488.95	126.70	713.60	402.90	584.80	159.10
12. Sample	22	485.21	507.25	142.10	729.30	415.60	597.80	161.69
13. Sample	22	499.78	522.15	156.50	748.50	428.40	623.80	164.54
14. Sample	22	514.81	545.20	165.40	767.10	440.80	641.80	167.84
15. Sample	22	530.91	570.35	174.50	789.50	453.20	664.30	170.30
16. Sample	22	546.85	593.15	186.50	804.50	469.60	675.60	170.31

f) Relative to Treatment Response=SD

Supplementary Table S4.19.: Normality assessment: beta-HCG changes over time in the Treatment Response=SD subgroup.

Normality assessment (serum b-HCG)Considered condition: Treatment Response=SD			
Variable	N	W	p
1. Sample	19	0.6084	<0.0001
2. Sample	19	0.6179	<0.0001
3. Sample	19	0.6222	<0.0001
4. Sample	19	0.6358	<0.0001
5. Sample	19	0.6452	<0.0001
6. Sample	19	0.6501	<0.0001
7. Sample	19	0.6580	<0.0001
8. Sample	19	0.6642	<0.0001
9. Sample	19	0.6774	<0.0001
10. Sample	19	0.6730	<0.0001
11. Sample	19	0.6774	<0.0001
12. Sample	19	0.6918	<0.0001
13. Sample	19	0.6780	<0.0001
14. Sample	19	0.6768	<0.0001
15. Sample	19	0.6850	<0.0001
16. Sample	19	0.6856	<0.0001

Supplementary TableS 4.20a.: ANOVA Friedman test and Kendall's coefficient of concordance: beta-HCG changes over time in the Treatment Response=SD subgroup.

Lack of normal distribution, performed ANOVA Friedman test.

ANOVA Friedman test and Kendall's coefficient of concordance(serum b-HCG)<br>Chi squared ANOVA(N = 19, df = 15) = 285.0000<br>p =<0.0001<br>Coefficient of concordance= 1.0000 r mean rank = 1.0000<br>Considered condition: Treatment Response=SD				
Variable	Mean Rank	Suma Rank	Mean	Standard Deviation
1. Sample	1	19	88.18	90.31
2. Sample	2	38	95.17	90.15
3. Sample	3	57	101.79	91.50
4. Sample	4	76	110.29	93.70
5. Sample	5	95	118.46	95.65
6. Sample	6	114	126.60	95.73
7. Sample	7	133	134.95	98.78
8. Sample	8	152	143.95	100.90
9. Sample	9	171	152.64	104.94
10. Sample	10	190	162.88	108.72
11. Sample	11	209	171.38	111.89
12. Sample	12	228	180.90	115.01
13. Sample	13	247	191.45	121.99
14. Sample	14	266	202.38	129.11
15. Sample	15	285	213.59	134.29
16. Sample	16	304	226.63	141.26

Supplementary Table S4.20b.: ANOVA Friedman test and Kendall's coefficient of concordance: beta-HCG changes over time in the Treatment Response=SD subgroup. Significant differences are highlighted.

**P-value**	1. Sample	2. Sample	3. Sample	4. Sample	5. Sample	6. Sample	7. Sample	8. Sample	9. Sample	10. Sample	11. Sample	12. Sample	13. Sample	14. Sample	15. Sample	16. Sample
1. Sample		1	1	1	1	0.1450	0.0123	0.0007	<0.0001	<0.0001	<0.0001	<0.0001	<0.0001	<0.0001	<0.0001	<0.0001
2. Sample	1		1	1	1	1	0.1450	0.0123	0.0007	<0.0001	<0.0001	<0.0001	<0.0001	<0.0001	<0.0001	<0.0001
3. Sample	1	1		1	1	1	1	0.1450	0.0123	0.0007	<0.0001	<0.0001	<0.0001	<0.0001	<0.0001	<0.0001
4. Sample	1	1	1		1	1	1	1	0.1450	0.0123	0.0007	<0.0001	<0.0001	<0.0001	<0.0001	<0.0001
5. Sample	1	1	1	1		1	1	1	1	0.1450	0.0123	0.0007	<0.0001	<0.0001	<0.0001	<0.0001
6. Sample	0.1450	1	1	1	1		1	1	1	1	0.1450	0.0123	0.0007	<0.0001	<0.0001	<0.0001
7. Sample	0.0123	0.1450	1	1	1	1		1	1	1	1	0.1450	0.0123	0.0007	<0.0001	<0.0001
8. Sample	0.0007	0.0123	0.1450	1	1	1	1		1	1	1	1	0.1450	0.0123	0.0007	<0.0001
9. Sample	<0.0001	0.0007	0.0123	0.1450	1	1	1	1		1	1	1	1	0.1450	0.0123	0.0007
10. Sample	<0.0001	<0.0001	0.0007	0.0123	0.1450	1	1	1	1		1	1	1	1	0.1450	0.0123
11. Sample	<0.0001	<0.0001	<0.0001	0.0007	0.0123	0.1450	1	1	1	1		1	1	1	1	0.1450
12. Sample	<0.0001	<0.0001	<0.0001	<0.0001	0.0007	0.0123	0.1450	1	1	1	1		1	1	1	1
13. Sample	<0.0001	<0.0001	<0.0001	<0.0001	<0.0001	0.0007	0.0123	0.1450	1	1	1	1		1	1	1
14. Sample	<0.0001	<0.0001	<0.0001	<0.0001	<0.0001	<0.0001	0.0007	0.0123	0.1450	1	1	1	1		1	1
15. Sample	<0.0001	<0.0001	<0.0001	<0.0001	<0.0001	<0.0001	<0.0001	0.0007	0.0123	0.1450	1	1	1	1		1
16. Sample	<0.0001	<0.0001	<0.0001	<0.0001	<0.0001	<0.0001	<0.0001	<0.0001	0.0007	0.0123	0.1450	1	1	1	1	

Supplementary Table S4.21.: Page test for trend: b-HCG changes over time in the Treatment Response=SD subgroup.

**Page Test for trend**	
Statistics L	28424
Statistics Z	16.8819
The one-sided p-value	<0.0001

Supplementary Table S4.22.: Descriptive statistics: b-HCG changes over time in the Treatment Response=SD subgroup.

Descriptive statistics (serum b-HCG)Considered condition: Treatment Response=SD								
Variable	N	Mean	Median	Minimum	Maximum	Lower Quartile	Upper Quartile	Standard Deviation
1. Sample	19	88.18	67.80	12.40	425.93	45.60	84.50	90.31
2. Sample	19	95.17	76.50	12.90	431.23	54.20	89.50	90.15
3. Sample	19	101.79	86.30	13.50	442.98	62.10	94.50	91.50
4. Sample	19	110.29	93.40	13.90	458.87	73.40	112.80	93.70
5. Sample	19	118.46	102.30	14.70	473.78	82.10	124.50	95.65
6. Sample	19	126.60	113.40	15.40	481.23	97.80	131.20	95.73
7. Sample	19	134.95	123.40	15.90	498.43	102.20	139.70	98.78
8. Sample	19	143.95	134.20	17.30	512.32	110.90	145.80	100.90
9. Sample	19	152.64	143.50	18.50	534.67	115.60	157.60	104.94
10. Sample	19	162.88	153.40	18.90	557.43	128.70	169.80	108.72
11. Sample	19	171.38	159.40	19.40	576.23	137.80	176.50	111.89
12. Sample	19	180.90	167.80	19.90	593.21	145.90	189.70	115.01
13. Sample	19	191.45	178.50	20.60	632.65	156.50	198.70	121.99
14. Sample	19	202.38	189.70	20.90	668.56	165.40	212.70	129.11
15. Sample	19	213.59	199.60	21.10	694.32	178.90	221.30	134.29
16. Sample	19	226.63	212.80	21.30	732.11	186.50	234.20	141.26

g) Relative to Treatment Response=PD

Supplementary Table S4.23.: Normality assessment: beta-HCG changes over time in the Treatment Response=PD subgroup.

Normality assessment (serum b-HCG)Considered condition: Treatment Response=PD			
Variable	N	W	p
1. Sample	21	0.9447	0.2690

Normality assessment (serum b-HCG)Considered condition: Treatment Response=PD			
Variable	N	W	p
2. Sample	21	0.9469	0.2969
3. Sample	21	0.9501	0.3427
4. Sample	21	0.9491	0.3275
5. Sample	21	0.9558	0.4350
6. Sample	21	0.9574	0.4651
7. Sample	21	0.9571	0.4591
8. Sample	21	0.9656	0.6352
9. Sample	21	0.9642	0.6052
10. Sample	21	0.9661	0.6471
11. Sample	21	0.9630	0.5793
12. Sample	21	0.9601	0.5178
13. Sample	21	0.9569	0.4562
14. Sample	21	0.9553	0.4267
15. Sample	21	0.9530	0.3868
16. Sample	21	0.9520	0.3711

Supplementary Table S4.24a.: ANOVA Friedman test and Kendall's coefficient of concordance: beta-HCG changes over time in the Treatment Response=PD subgroup.

Lack of normal distribution, performed ANOVA Friedman test.

ANOVA Friedman test and Kendall's coefficient of concordance(serum b-HCG)<br>Chi squared ANOVA(N = 21, df = 15) = 315.0000<br>p =<0.0001<br>Coefficient of concordance= 1.0000 r mean rank = 1.0000<br>Considered condition: Treatment Response=PD				
Variable	Mean Rank	Suma Rank	Mean	Standard Deviation
1. Sample	1	21	339.07	111.62
2. Sample	2	42	353.36	114.64
3. Sample	3	63	367.77	116.08
4. Sample	4	84	381.20	117.37
5. Sample	5	105	397.40	119.47
6. Sample	6	126	413.45	121.95
7. Sample	7	147	427.66	124.39
8. Sample	8	168	446.33	127.99
9. Sample	9	189	461.28	130.38
10. Sample	10	210	477.94	133.69
11. Sample	11	231	492.19	136.50
12. Sample	12	252	507.73	139.15
13. Sample	13	273	522.62	142.27
14. Sample	14	294	537.38	146.24
15. Sample	15	315	552.91	149.85
16. Sample	16	336	568.04	151.34

Supplementary Table S4.24b.: ANOVA Friedman test and Kendall's coefficient of concordance: beta-HCG changes over time in the Treatment Response=PD subgroup. Significant differences are highlighted.

**P-value**	1. Sample	2. Sample	3. Sample	4. Sample	5. Sample	6. Sample	7. Sample	8. Sample	9. Sample	10. Sample	11. Sample	12. Sample	13. Sample	14. Sample	15. Sample	16. Sample
1. Sample		1	1	1	0.7776	0.0800	0.0053	0.0002	<0.0001	<0.0001	<0.0001	<0.0001	<0.0001	<0.0001	<0.0001	<0.0001
2. Sample	1		1	1	1	0.7776	0.0800	0.0053	0.0002	<0.0001	<0.0001	<0.0001	<0.0001	<0.0001	<0.0001	<0.0001
3. Sample	1	1		1	1	1	0.7776	0.0800	0.0053	0.0002	<0.0001	<0.0001	<0.0001	<0.0001	<0.0001	<0.0001
4. Sample	1	1	1		1	1	1	0.7776	0.0800	0.0053	0.0002	<0.0001	<0.0001	<0.0001	<0.0001	<0.0001
5. Sample	0.7776	1	1	1		1	1	1	0.7776	0.0800	0.0053	0.0002	<0.0001	<0.0001	<0.0001	<0.0001
6. Sample	0.0800	0.7776	1	1	1		1	1	1	0.7776	0.0800	0.0053	0.0002	<0.0001	<0.0001	<0.0001
7. Sample	0.0053	0.0800	0.7776	1	1	1		1	1	1	0.7776	0.0800	0.0053	0.0002	<0.0001	<0.0001
8. Sample	0.0002	0.0053	0.0800	0.7776	1	1	1		1	1	1	0.7776	0.0800	0.0053	0.0002	<0.0001
9. Sample	<0.0001	0.0002	0.0053	0.0800	0.7776	1	1	1		1	1	1	0.7776	0.0800	0.0053	0.0002
10. Sample	<0.0001	<0.0001	0.0002	0.0053	0.0800	0.7776	1	1	1		1	1	1	0.7776	0.0800	0.0053
11. Sample	<0.0001	<0.0001	<0.0001	0.0002	0.0053	0.0800	0.7776	1	1	1		1	1	1	0.7776	0.0800
12. Sample	<0.0001	<0.0001	<0.0001	<0.0001	0.0002	0.0053	0.0800	0.7776	1	1	1		1	1	1	0.7776
13. Sample	<0.0001	<0.0001	<0.0001	<0.0001	<0.0001	0.0002	0.0053	0.0800	0.7776	1	1	1		1	1	1
14. Sample	<0.0001	<0.0001	<0.0001	<0.0001	<0.0001	<0.0001	0.0002	0.0053	0.0800	0.7776	1	1	1		1	1
15. Sample	<0.0001	<0.0001	<0.0001	<0.0001	<0.0001	<0.0001	<0.0001	0.0002	0.0053	0.0800	0.7776	1	1	1		1
16. Sample	<0.0001	<0.0001	<0.0001	<0.0001	<0.0001	<0.0001	<0.0001	<0.0001	0.0002	0.0053	0.0800	0.7776	1	1	1	

Supplementary Table S4.25.: Page test for trend: b-HCG changes over time in the Treatment Response=PD subgroup.

**Page Test for trend**	
Statistics L	31416
Statistics Z	17.748239
The one-sided p-value	<0.0001

Supplementary Table S4.26.: Descriptive statistics: b-HCG changes over time in the Treatment Response=PD subgroup.

Descriptive statistics (serum b-HCG)Considered condition: Treatment Response=PD								
Variable	N	Mean	Median	Minimum	Maximum	Lower Quartile	Upper Quartile	Standard Deviation
1. Sample	21	339.07	356.40	37.80	543.80	287.90	423.50	111.62
2. Sample	21	353.36	376.30	43.20	562.10	299.30	439.70	114.64
3. Sample	21	367.77	389.50	54.80	579.20	310.60	452.70	116.08
4. Sample	21	381.20	402.60	64.30	595.20	322.40	462.90	117.37
5. Sample	21	397.40	420.40	76.50	619.30	335.80	476.50	119.47
6. Sample	21	413.45	438.40	87.60	638.70	347.60	493.40	121.95
7. Sample	21	427.66	451.20	94.50	651.20	358.90	512.80	124.39
8. Sample	21	446.33	462.40	109.80	668.30	367.80	531.90	127.99
9. Sample	21	461.28	476.50	116.70	681.30	381.70	546.70	130.38
10. Sample	21	477.94	487.60	127.80	695.20	398.70	564.30	133.69
11. Sample	21	492.19	496.70	132.70	713.60	412.90	584.80	136.50
12. Sample	21	507.73	515.80	139.80	729.30	432.70	597.80	139.15
13. Sample	21	522.62	527.80	145.60	748.50	452.30	623.80	142.27
14. Sample	21	537.38	546.70	153.20	767.10	462.80	641.80	146.24
15. Sample	21	552.91	576.30	159.80	789.50	472.70	664.30	149.85
16. Sample	21	568.04	598.70	165.40	804.50	478.50	675.60	151.34

5. Comparison between Sample 16 and Sample 1 across study groups

We calculated the difference between the final and initial b-HCG concentration for every patient in order to see whether the change is affected by a set of variables.

a) Liver Tumor Burden

Supplementary Table S5.1.: Normality assessment: the difference in b-HCG levels over 48 months in Liver Tumor Burden=10% subgroup.

Liver Tumor Burden = up to 10%; Normality assessment (serum b-HCG)			
Variable	N	W	p
the difference between 16 and 1	22	0.88818	0.0174

Supplementary Table S5.2.: Normality assessment: the difference in b-HCG levels over 48 months in Liver Tumor Burden=10-25% subgroup.

Liver Tumor Burden = 10-25%; Normality assessment (serum b-HCG)			
Variable	N	W	p
the difference between 16 and 1	18	0.8072	0.0019

Supplementary Table S5.3a.: Mann-Whitney U Test (with continuity correction): the difference in b-HCG levels over 48 months vs Liver Tumor Burden.

Due to the lack of conformity with the normal distribution – Manna-Whitney U Test.

Mann-Whitney U Test (with continuity correction) (serum b-HCG) Vs variable: Liver Tumor Burden Highlighted values are significant, with 2-sided p<0.05						
Variable	Sum.rank 10%	Sum.rank 10-25%	U	Z	p	Z corrected
the difference between 16 and 1	307	513	54	-3.9012	<0.0001	-3.90143

Supplementary Table S5.3b.: Mann-Whitney U Test (with continuity correction): the difference in b-HCG levels over 48 months vs Liver Tumor Burden.

Mann-Whitney U Test (with continuity correction) (serum b-HCG) Vs variable: Liver Tumor Burden Highlighted values are significant, with 2-sided p<0.05				
Variable	p	N 10%	N 10-25%	2*1-sided exact. p
the difference between 16 and 1	<0.0001	22	18	<0.0001

Supplementary Table S5.4a.: Descriptive statistics: the difference in b-HCG levels over 48 months in the Liver Tumor Burden=10% subgroup

Liver Tumor Burden = up to 10%; Descriptive statistics (serum b-HCG)						
Variable	N	Mean	Median	Minimum	Maximum	Lower Quartile
the difference between 16 and 1	22	139.56	146.65	8.90	306.18	113.70

Supplementary Table S5.4b.: Descriptive statistics: the difference in b-HCG levels over 48 months in the Liver Tumor Burden=10% subgroup.

Liver Tumor Burden = up to 10%; Descriptive statistics (serum b-HCG)		
Variable	Upper Quartile	Standard Deviation
the difference between 16 and 1	166.40	58.78

Supplementary Table S5.5a.: Descriptive statistics: the difference in b-HCG levels over 48 months in the Liver Tumor Burden=10-25% subgroup.

Liver Tumor Burden = 10-25%; Descriptive statistics (serum b-HCG)						
Variable	N	Mean	Median	Minimum	Maximum	Lower Quartile
the difference between 16 and 1	18	242.71	267.00	52.70	311.10	239.70

Supplementary Table S5.5b.: Descriptive statistics: the difference in b-HCG levels over 48 months in the Liver Tumor Burden=10-25% subgroup.

Liver Tumor Burden = 10-25%; Descriptive statistics (serum b-HCG)		
Variable	Upper Quartile	Standard Deviation
the difference between 16 and 1	281.10	66.50

b) Grading

Supplementary Table S5.6.: Normality assessment: the difference in b-HCG levels over 48 months in Grading=G1 subgroup.

grading=G1; Normality assessment (serum b-HCG)			
Variable	N	W	p
the difference between 16 and 1	18	0.9259	0.1645

Supplementary Table S5.7.: Normality assessment: the difference in b-HCG levels over 48 months in Grading=G2 subgroup.

grading=G2; Normality assessment (serum b-HCG)			
Variable	N	W	p
the difference between 16 and 1	22	0.8945	0.0231

Supplementary Table S5.8a.: Mann-Whitney U Test (with continuity correction): the difference in b-HCG levels over 48 months vs Grading.

Lack of normal distribution, performed Mann-Whitney U Test with continuity correction.

Mann-Whitney U Test (with continuity correction) (serum b-HCG)<br>Vs variable: grading<br>Highlighted values are significant, with 2-sided p<0.05						
Variable	Sum.rank G1	Sum.rank G2	U	Z	p	Z corrected
the difference between 16 and 1	261	559	90	-2.9225	0.0035	-2.9227

Supplementary Table S5.8b.: Mann-Whitney U Test (with continuity correction): the difference in b-HCG levels over 48 months vs Grading.

Mann-Whitney U Test (with continuity correction) (serum b-HCG)<br>Vs variable: grading<br>Highlighted values are significant, with 2-sided p<0.05				
Variable	p	N G1	N G2	2*1-sided exact. p
the difference between 16 and 1	0.0035	18	22	0.0028

Supplementary Table S5.9a.: Descriptive statistics: the difference in b-HCG levels over 48 months in the Grading=G1 subgroup.

grading=G1; Descriptive statistics (serum b-HCG)						
Variable	N	Mean	Median	Minimum	Maximum	Lower Quartile
the difference between 16 and 1	18	143.23	146.90	8.90	306.18	113.70

Supplementary Table S5.9b.: Descriptive statistics: the difference in b-HCG levels over 48 months in the Grading=G1 subgroup.

grading=G1; Descriptive statistics (serum b-HCG)		
Variable	Upper Quartile	Standard Deviation
the difference between 16 and 1	167.20	67.67

Supplementary Table S5.10a.: Descriptive statistics: the difference in b-HCG levels over 48 months in the Grading=G2 subgroup.

grading=G2; Descriptive statistics (serum b-HCG)						
Variable	N	Mean	Median	Minimum	Maximum	Lower Quartile
the difference between 16 and 1	22	220.95	256.25	52.70	311.10	161.40

Supplementary Table S5.10b.: Descriptive statistics: the difference in b-HCG levels over 48 months in the Grading=G2 subgroup.

grading=G2; Descriptive statistics (serum b-HCG)		
Variable	Upper Quartile	Standard Deviation
the difference between 16 and 1	278.20	74.14

c) Treatment Response

Supplementary Table S5.11.: Normality assessment: the difference in b-HCG levels over 48 months in Treatment Response=SD subgroup.

Treatment Response=SD; Normality assessment (serum b-HCG)			
Variable	N	W	p
the difference between	19	0.8775	0.0195

Treatment Response=SD; Normality assessment (serum b-HCG)			
Variable	N	W	p
16 and 1			

Supplementary Table S5.12.: Normality assessment: the difference in b-HCG levels over 48 months in Treatment Response=PD subgroup.

Treatment Response=PD; Normality assessment (serum b-HCG)			
Variable	N	W	p
the difference between 16 and 1	21	0.8745	0.0116

Supplementary Table S5.13a.: Mann-Whitney U Test (with continuity correction): the difference in b-HCG levels over 48 months vs Treatment Response.

Mann-Whitney U Test (with continuity correction) (serum b-HCG) Vs variable: Treatment Response Highlighted values are significant, with 2-sided p<0.05						
Variable	Sum.rank SD	Sum.rank PD	U	Z	p	Z corrected
the difference between 16 and 1	263	557	73	-3.4126	0.0006	-3.4127

Supplementary Table S5.13b.: Mann-Whitney U Test (with continuity correction): the difference in b-HCG levels over 48 months vs Treatment Response.

Mann-Whitney U Test (with continuity correction) (serum b-HCG) Vs variable: Treatment Response Highlighted values are significant, with 2-sided p<0.05				
Variable	p	N SD	N PD	2*1-sided exact. p
the difference between 16 and 1	0.0006	19	21	0.0004

Supplementary Table S5.14a.: Descriptive statistics: the difference in b-HCG levels over 48 months in the Treatment Response=SD subgroup.

Treatment Response=SD; Descriptive statistics (serum b-HCG)						
Variable	N	Mean	Median	Minimum	Maximum	Lower Quartile
the difference between 16 and 1	19	138.46	147.50	8.90	306.18	111.70

Supplementary Table S5.14b.: Descriptive statistics: the difference in b-HCG levels over 48 months in the Treatment Response=SD subgroup.

Treatment Response=SD; Descriptive statistics (serum b-HCG)		
Variable	Upper Quartile	Standard Deviation
the difference between 16 and 1	166.40	62.16

Supplementary Table S5.15a.: Descriptive statistics: the difference in b-HCG levels over 48 months in the Treatment Response=PD subgroup.

Treatment Response=PD; Descriptive statistics (serum b-HCG)						
Variable	N	Mean	Median	Minimum	Maximum	Lower Quartile
the difference between 16 and 1	21	228.98	260.70	52.70	311.10	181.70

Supplementary Table S5.15b.: Descriptive statistics: the difference in b-HCG levels over 48 months in the Treatment Response=PD subgroup.

Treatment Response=PD; Descriptive statistics (serum b-HCG)		
Variable	Upper Quartile	Standard Deviation
the difference between 16 and 1	278.20	71.34

d) Patient's age

Supplementary Table S5.16.: Normality assessment: the difference in b-HCG levels over 48 months relative to patient's age.

Normality assessment (serum b-HCG)			
Variable	N	W	p
the difference between 16 and 1	40	0.9416	0.0391
age	40	0.9582	0.1464

Supplementary Table S5.17.: Spearman rank correlation coefficient: the difference in b-HCG levels over 48 months vs patient age.

Due to the lack of conformity with the normal distribution – Spearman rank correlation coefficient.

Spearman rank correlation coefficient. (serum b-HCG) Correlation coefficients are significant, when p <0.05				
Variable Pair	N	R Spearman	t(N-2)	p
age & the difference between 16 and 1	40	-0.0069	-0.0423	0.9665

6. Logistic regression analysis – predictive power of Initial b-HCG concentrations on the occurrence of Progressive Disease

Supplementary Table S6.1.: Logistic regression analysis of Initial β-hCG concentration for predicting Progressive Disease.

	b coeff.	b error	-95% CI	+95% CI	Wald stat.	p-value	odds ratio	-95% CI	+95% CI
intercept	-3.2841	0.9822	-5.2093	-1.3590	11.1789	0.0008	0.0375	0.0055	0.2569
1st measure	0.0171	0.0047	0.0079	0.0264	13.1962	0.0003	1.0173	1.0079	1.0268

Supplementary Table S6.2.: Classification table for the logistic regression model.

Classification		Observed value	
		1	0
Predicted value	1	19	1
	0	2	18

Cut-off line	0.5
% correct	92.5%
Sensitivity (% correct 1)	90.48%
-95% CI	69.62%
+95% CI	98.83%
Specificity (% correct 0)	94.74%
-95% CI	73.97%
+95% CI	99.88%

Supplementary Table S6.3.: Model Fit Statistics and Predictive Performance for Logistic Regression Analysis.

**Likelihood ratio test**	
Log Likelihood	-12.1224
-2 Log Likelihood	24.2448
Log Likelihood (intercept)	-27.6759
-2 Log Likelihood (intercept)	55.3517
Chi-square statistic	31.1069
Degrees of freedom	1
p-value	<0.0001
AIC - Akaike criterion	26.2448
AICc - corrected Akaike criterion	26.3501
BIC - Bayesian criterion	27.9337
Pseudo R2	0.5620
R2(Nagelkerke)	0.7213
R2(Coxa-Snella)	0.5405
**Hosmer-Lemeshow test**	
Chi-square statistic	17.0679
Degrees of freedom	8
p-value	0.0294
**ROC curves (DeLong's method)**	
AUC	0.9173
SE(AUC)	0.0559

-95% CI	0.8077
+95% CI	1
Z statistic	4.5095
p-value	<0.0001
Cut-off line	0.5931
